# Quantifying the Extent to Which Junior Performance Predicts Senior Performance in Olympic Sports: A Systematic Review and Meta-analysis

**DOI:** 10.1007/s40279-023-01906-0

**Published:** 2023-09-07

**Authors:** Michael Barth, Arne Güllich, Brooke N. Macnamara, David Z. Hambrick

**Affiliations:** 1 Department of Sport Science, University of Innsbruck, Fürstenweg 176, 6020 Innsbruck, Austria; 2 Department of Sports Science, RPTU Kaiserslautern, Erwin-Schrödinger-Straße 57, 67663 Kaiserslautern, Germany; 3 Department of Psychological Sciences, Case Western Reserve University, 11220 Bellflower Road, Cleveland, OH 44106 USA; 4 Department of Psychology, Michigan State University, 316 Physics Road, East Lansing, MI 48825 USA

## Abstract

**Background:**

To what extent does junior athletic performance predict senior athletic performance (i.e., in the highest, open-age category)? This question is the subject of a lively debate in the literature. Following traditional theories of giftedness and expertise, some researchers and practitioners have proposed that a high level of junior performance is a prerequisite for the development of a high level of later senior performance. Sceptics of this view hold that junior performance has limited predictive value for later senior performance, pointing to empirical evidence indicating that predictors (e.g., participation patterns) of junior performance and of senior performance differ. The straightforward way to resolve this controversy empirically is to test the correlation between junior and senior performance.

**Objective:**

To provide robust and generalizable evidence on this issue, we performed a systematic review and meta-analysis of relevant studies. The aim was to quantify the overall correlation between junior and senior performance and then test whether correlations vary across junior age categories and subsamples (e.g., types of sports).

**Methods:**

A systematic literature search was conducted in SPORTDiscus, Eric, ProQuest, PsychInfo, PubMed, Scopus, WorldCat, and Google Scholar from 27 January through 30 April 2022. We searched for original studies that recorded athletes’ junior and senior performance longitudinally and included measures of association between junior and senior performance. Quality of evidence was evaluated using the Mixed Methods Appraisal Tool version for nonrandomized studies.

**Results:**

The search yielded *k* = 129 effect sizes from *N* = 13,392 athletes from a wide range of Olympic sports, 62% male and 38% female, from 2006 to 2021. Four central findings emerged: (1) Overall, the meta-analytic pooled correlation between junior and senior performance was $\overline{r }$ = 0.148. That is, junior performance explained only 2.2% of the reliable variance in senior performance. (2) The finding was robust across types of sports, sexes, wider or narrower performance ranges, national or international samples, and binary or continuous performance measures. (3) Effects varied across junior age categories: the younger the junior age category, the lower the correlation between junior and senior performance, with $\overline{{r} }$ ranging from $\overline{{r} }$ = − 0.052 to $\overline{{r} }$ = 0.215. That is, across junior age categories, junior performance explained 0–4.6% of the reliable variance in senior performance. (4) The quality of primary studies was high.

**Discussion:**

The results suggest that junior performance has very little, if any, predictive value for senior performance. The findings run counter to claims from traditional theories of both giftedness and expertise. From an applied perspective, talent selection typically begins around puberty or younger—age ranges where youth performance is uncorrelated or negatively correlated with later senior performance. The evidence presented here raises serious questions about the use of junior performance for talent selection purposes.

A PRISMA-P protocol was registered at https://osf.io/gck4a/.

**Supplementary Information:**

The online version contains supplementary material available at 10.1007/s40279-023-01906-0.

## Key Points

**Table:** 

Junior performance has very little predictive value for later senior performance.
The finding is robust across different populations (e.g., types of sports, sexes, geographical regions).
Effects vary across junior age categories: The younger the junior age category, the lower the correlation between junior and senior performance.

## Introduction

“Talent” in sports can be understood as a youth athlete’s potential for the long-term development of a high performance level in future elite sports. The belief in a youth athlete’s talent often leads to expanded investments by the athlete, parents, and talent promotion programs.

Talent identification in sports often begins around puberty or younger [1–3] and includes the forecasting of the youth athlete’s future performance development. Attempts to forecast human performance development are common in multiple fields, such as school entry tests, admission to music schools and conservatoria, admission to college or university, and job application interviews [4–6], although long-term prognostic validity is often poor [7–15]. In sports, forecasts of future performance are the basis of admission to talent promotion programs, including federations’ youth squads and selection teams, youth sport academies, and centers of excellence.

Although more complex talent identification systems have been developed in the recent years [2], most talent selection procedures have one fundamental idea in common: predicting future performance by past or current performance. Here, we investigate the extent to which earlier performance predicts later performance in sports. More specifically, we ask: to what extent does *junior* athletic performance predict *senior* athletic performance?

Although junior performance is a predictor commonly used in talent selection, a lively debate exists in the sports science and medicine literature. Proponents of using junior performance to predict senior performance assume a high level of performance in youth is a prerequisite for the development of a high level of performance in adulthood. This view follows from theories of giftedness and expertise (e.g., [4, 16–18]) and is in line with claims made by numerous applied researchers and practitioners (e.g., [19–26]). It also corresponds with sport policies and practices in terms of establishment of continental and world championships, festivals, and circuits for competitors as young as 11–15 years old [27].

Sceptics of this practice point to several empirical observations. First, predictors of junior performance and senior performance are different and in some cases opposite [28, 29]. For example, highly successful junior athletes reach performance milestones rapidly, whereas highly successful senior athletes progressed more gradually in their early years. Furthermore, early biological maturation (puberty, growth spurt) [30] facilitates junior performance, but not senior performance. Likewise, youth athletes who are born early within their age year have a performance advantage in adolescence (relative age effect, RAE, e.g., [31]), which diminishes or is even reversed by adulthood [32–34].

Second, multiple factors develop at different timescales and rates within individuals and between individuals (e.g., coaching, practice, skill acquisition, psychosocial development, or social support). Third, predictors of performance do not necessarily predict persistence: many youth athletes, including highly successful juniors, withdraw from competition sports before adulthood [35]. Finally, at junior-level championships, athletes compete within a single, typically 2-year age group, whereas at senior-level championships, athletes compete with peers from a wider age range, often from late teens to late 30s [27].

In short, the popular view that the best predictor of later performance is earlier performance notwithstanding (e.g., [4]), there is ample reason to question the validity of using junior performance to predict senior performance [28, 29]. Fortunately, there is a straightforward empirical test to resolve this controversy: The analysis of the correlation between earlier and later performance—in our case, the correlation between junior and senior performance.

If junior performance is a strong predictor of senior performance, there should be strong positive correlations between junior and senior performance. If junior performance is not a strong predictor of senior performance, there should be weak or null correlations between junior and senior performance. The former finding would support the use of junior performance for talent selection; the latter finding would indicate this practice may be misguided.

To test these two competing hypotheses, we performed a systematic review and meta-analysis examining the relationship between junior performance and senior performance. Compiling all available relevant empirical evidence, we estimated the overall magnitude of the correlation between junior performance and senior performance, and also tested whether findings vary across junior age categories and different subsamples (e.g., type of sports, sex).^1^

## Methods

**Fig. 1:**
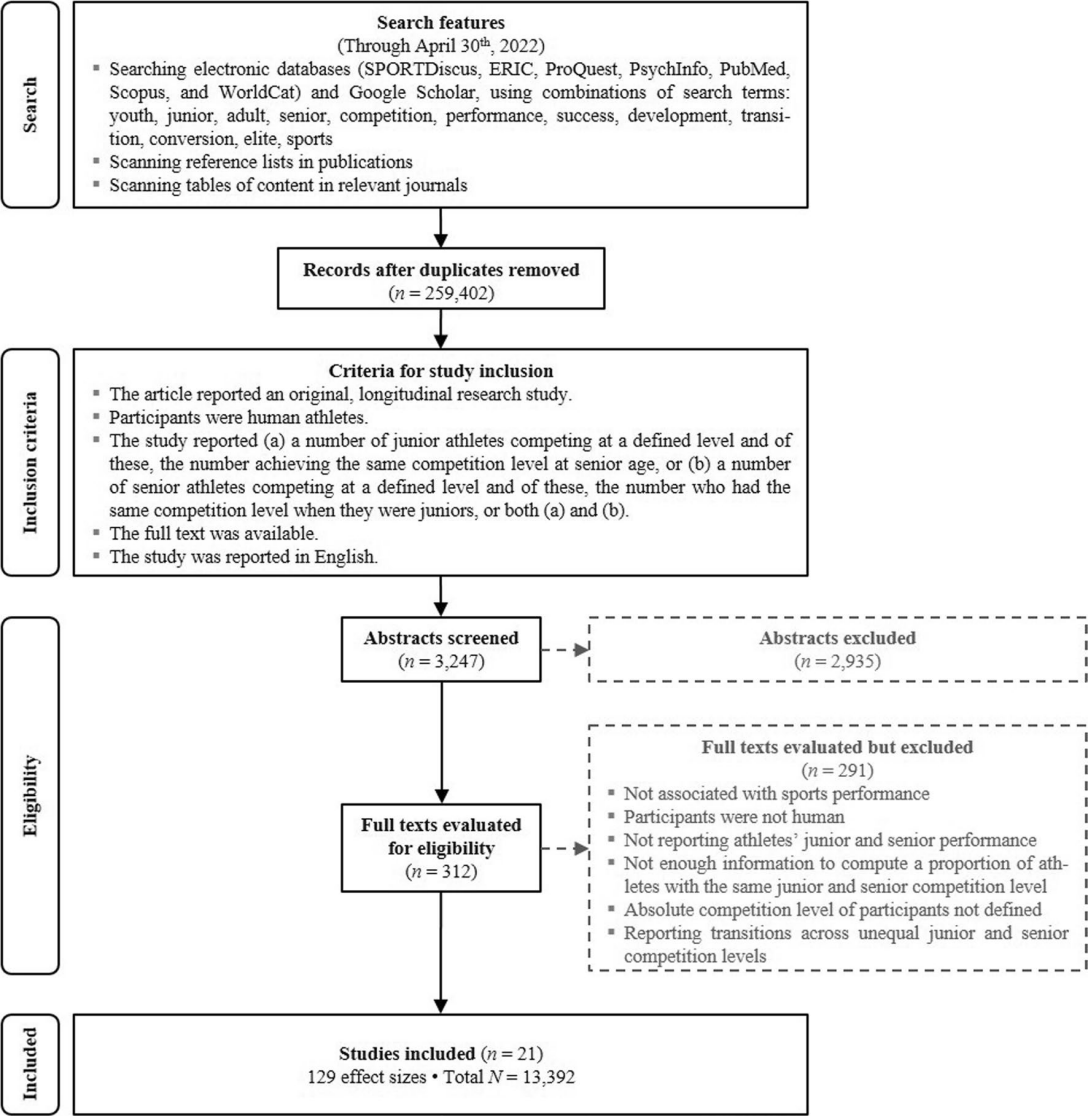
Flow diagram of the literature search and study coding

The study search and selection procedures were guided by the PRISMA 2020 statement (Preferred Reporting Items for Systematic Reviews and Meta-Analyses [36], a PRISMA-P [37] protocol was registered at https://osf.io/gck4a/). We searched for original studies that recorded athletes’ junior and senior performance longitudinally and reported a measure of association between junior and senior performance or data needed to compute that association. Figure 1 shows the flowchart of the major steps of the literature search and screening, which was conducted from 27 January through 30 April 2022.

### Sample

The search and selection yielded a total of *k* = 129 effect sizes from *N* = 13,392 athletes, 61.9% male and 38.1% female, reported in 21 study reports from 2006 to 2021. There were additional effect sizes because individual studies reported associations between junior and senior performance for different subsamples (e.g., male and female, different sports or disciplines) and for multiple junior age categories (see below).

Table 1 presents characteristics of the total sample. The sample included athletes from a wide range of sports of the Olympic Games. About three-quarters of the athletes (72.1%) were from international samples (athletes from multiple countries, e.g., participants at the Olympic Games, world or continental championships, or athletes listed in international rankings) and about one-quarter (27.9%) were from national samples (athletes from one country, respectively), all from Western European countries. The characteristics and corresponding measures of association between junior and senior performance of the relevant studies are provided in the electronic supplementary material (ESM) 1.

**Table 1:** Sample characteristics and subsample sizes (number of athletes)

Subsample	*N*
Year of study report	
Until 2009	958
2010–2014	5943
2015–2021	6491
Sex	
Male	7590
Female	4676
Male and female	1126
Types of sport by the task in competition^a^	
Cgs sports (e.g., athletics, rowing, swimming, ski racing)	5803
Game sports (e.g., basketball, soccer, tennis)	6310
Combat sports (e.g., fencing, judo, taekwondo)	158
Artistic composition sports (e.g., artistic gymnastic, figure skating)	35
Other sports (e.g., modern pentathlon, ski jumping)/multiple sports	1086
Individual and team sports^b^	
Individual sports (e.g., athletics, judo, race cycling, swimming, tennis)	9623
Team sports (e.g., basketball, hockey, rugby, soccer, volleyball)	2593
Individual sports and team sports	1176
Region^c^	
International samples (e.g., participants at world or continental championships)	9650
National samples (from just one country, respectively)	3742
Junior age category^d^	
Junior A	5576
Junior B	2761
Junior C	4312
Junior D	743

^a^Types of sports were categorized on the basis of the task in competition [38]: *Cgs* (performance is measured in centimeters, grams, or seconds), *game*, *combat*, *artistic composition*, and *others/multiple* sports (sports meeting none or multiple criteria of the aforementioned sports types or samples involving different types of sports)

^b^*Individual sports* (individual athletes compete with one another), *team sports* (teams compete with one another)

^c^*International samples* (athletes from multiple countries), *national samples* (athletes from just one country, respectively)

^d^*Junior A, Junior B, Junior C, Junior D* junior age categories, with Junior A being the oldest and Junior D the youngest age category within each sport

The primary studies either reported athletes’ ages as the sample mean and standard deviation or the minimum and maximum ages. Across studies, the sample-weighted mean age as a senior was 25.7 years, the sample-weighted mean minimum age as a senior was 20.2 years, and the sample-weighted mean maximum age was 36.3 years. Athlete performance data were collected from public records (official championship results or ranking lists) for 11,742 athletes and by athlete questionnaires or interviews for 1650 athletes.

### Data Extraction, Performance Levels, and Junior Age Categories

Each study was coded for (1) author(s) and year, (2) publication status, (3) method of data collection, (4) sample characteristics (country, sport, sex, age, junior age categories involved, range of competition levels, number of athletes), and (5) the correlation between junior performance and senior performance.

Performance differences between athletes were determined in the primary studies by either dichotomous classification (*k* = 39) or continuous performance measures (*k* = 90). Dichotomous performance measures recorded whether an athlete did or did not achieve a defined competition level, respectively at junior and senior age, such as (1) playing in the highest national league; (2) nomination for a national selection team or talent promotion program; or (3) participating or achieving a top-ten placing at major international championships. For these dichotomous performance variables, the corresponding measure of association between junior and senior performance is the *φ* coefficient.

Continuous performance measures, both at junior and senior competitions, included (1) athletes’ championship level (world, continental, national, regional level) and placing at that championship; (2) placings in official international rankings (e.g., Association of Tennis Professionals ranking list); (3) an athlete’s number of international junior championships, games or minutes played at an international championship; (4) swimmers’ peak swim time; or (5) basketball players’ composite score from official FIBA game statistics (including minutes played, scored points, assists, rebounds, steals, and blocks). For these continuous performance variables, the corresponding measure of association between junior and senior performance is the correlation coefficient *r*.

Thus, the two approaches allowed us to investigate (1) the extent to which being within a defined junior performance range (e.g., being among junior international 1st–10th) predicts achieving a defined senior performance range (e.g., being among senior international 1st–10th), and (2) the extent to which individual differences in athlete’s competition level and exact rank at junior age (e.g., national 1st, 2nd, 3rd, …, 10th, international 1st, 2nd, 3rd, …, 10th) predict their individual differences in competition level and rank at senior age (national 1st, 2nd, 3rd, …, 10th, international 1st, 2nd, 3rd, …, 10th). This also enabled us to compare whether the approaches differ in predictive power.

Furthermore, we considered the range of the junior and senior performance levels examined in each study because wider performance ranges (e.g., regional level to international level) may yield larger associations, whereas narrower performance ranges (e.g., only national to international level) may yield smaller associations (restriction of range, see [39]). In our analysis, all primary studies involved senior international and national-level athletes and *k* = 32 also involved lower, regional-level athletes. We thus distinguished between “narrow range” samples (national to international championship level, *k* = 97) and “wide range” samples (regional to international championship level, *k* = 32) (see also [28]).

Junior age categories are defined by the regulations of the international sport federation for each sport. However, the labels for the different junior age groups differ by sport and country. For this report, we uniformly use the following labels: Junior A, Junior B, Junior C, and Junior D, where Junior A is the oldest junior age category (in most sports 17–18 or 18–19 years), Junior B is two years younger than Junior A, Junior C is 2 years younger than Junior B, and Junior D is the youngest age category within each sport (in most sports 11–12 or 12–13 years). The sample included *k* = 62 effect sizes for the association between Junior A performance and senior performance, *k* = 45 effect sizes for the association between Junior B performance and senior performance, *k* = 13 effect sizes for Junior C performance and senior performance, and *k* = 9 effect sizes for Junior D performance and senior performance.

### Meta-analytic Approach

All analyses were performed using the publicly available R environment, version 4.1.3. Effect sizes are expressed as *r*. Correlations reported as *φ* were converted to *r*. To synthesize effect sizes across studies, all *r* were converted to Fisher’s *z* (using the function “transf.rtoz,” package metafor 3.4.0). After estimating the meta-analytic models, we reconverted the meta-analytic mean Fisher’s $\overline{{z} }$ metrics to pooled correlation coefficients $\overline{{r} }$ (function “transf.ztor,” metafor 3.4.0 package).

We searched for outliers, defined as a Fisher’s *z* whose residuals had *z* scores > 3 [28]. No more than two outliers were identified within any subsample. Outlying values were excluded from subsequent analyses.

Independence of effect sizes is a crucial assumption of conventional meta-analytic procedures. We assumed that athletes involved in different studies, male and female athletes, and athletes from different sports were from independent samples. However, in several studies, athletes’ performance data were collected from various junior age categories within sexes and sports, where the extent of sample independence or dependence was not always clear from the primary reports. Becker [40] described four central approaches for dealing with partially dependent samples. The four approaches are: (1) treating data as independent, (2) combining across different outcomes, (3) creating independent data sets, and (4) modeling dependence.

Given the nature of the data in our synthesis, we decided to apply the following approaches to estimate the overall pooled correlation and compare the respective models: (1) assuming independence of all samples; (2) combining across different effect sizes; for this approach, Cheung and Chan [41] described a sample-wise adjustment by individual effect size (see Cheung and Chan’s R script “MADependentESFunctions.r” in [41]); (3) modeling dependence by application of three-level modeling using cluster-robust variance estimation (RVE, metaphor 3.4.0 package, functions “rma.mv” and “robust” [42]), and with using a structural equation modeling approach (metaSem 1.2.5.1 package, function “meta3”).

Pooled effects were similar across the different models, 0.145 ≤ $\overline{{r} }$ ≤ 0.151 (model differences 0.597 ≤ *p* ≤ 0.955). Following Becker’s recommendations, the most complete and accurate portrayal of the effects of dependence requires the modeling of this dependency. Therefore, we used three-level RVE modeling for subsequent moderator analyses. We estimated the overall effect of junior performance on senior performance by conducting a random effects meta-analysis (*k* = 129) and then employed mixed-effect models to analyze whether defined subsample characteristics moderated effects. All subsamples are provided separately in the ESM 1.

### Moderator Analyses

We investigated whether subsamples differed in effect sizes using mixed-effects models with Wald’s *F* [43]. For all moderator analyses, we used the rule of thumb that *k* ≥ 5 is required for each subgroup [44].

We tested for effects of six moderators:

1. Type of performance measure: dichotomous versus continuous.
2. Range of performance levels: narrow versus wide.
3. Gender: male versus female.
4. Junior age category: Junior A, Junior B, Junior C, and Junior D.
5. Type of sports: cgs, game, and combat sports; and individual versus team sports. Unlike in individual sports, in team sports a relatively low-performing individual athlete may be successful by playing on a high-performing team, whereas a high-performing player may be unsuccessful by playing on a low-performing team.
6. Region: national samples, i.e., athletes from just one nation (e.g., participants at a national championship), versus international samples, i.e. athletes from multiple nations (e.g., participants at Olympic Games, world, and continental championships). The latter may show a higher and more homogeneous performance level than the former.

All moderator analyses (except for junior age category) were performed controlling for junior age category. All hypothesis testing was two-tailed, and *p* < 0.05 was considered statistically significant. Following Cohen [45], we considered *r* ≈ 0.10 a “small” effect, *r* ≈ 0.30 a “medium” effect, and *r* ≈ 0.50 a “large” effect.

### Quality Assessment and Risk of Bias

We assessed the quality of the primary studies by the Mixed Methods Appraisal Tool (MMAT) version for nonrandomized studies [46]. Given that we analyzed empirical quantitative studies only, the two preliminary screening questions of the MMAT pertaining to qualitative studies were not relevant here. Furthermore, the fifth MMAT item—whether an intervention was administered as intended—was not applicable, as the primary studies did not involve interventions. Thus, we assessed the quality of the primary studies by the following four MMAT criteria (see ESM 2): representativeness, appropriateness of measurements, completeness of outcome data, and consideration of confounders. All studies were independently assessed by the first and the second author; inter-rater reliability was excellent (Cohen’s *κ* = 95%). In addition, we examined whether studies collecting athletes’ performance by document analyses (of data from official public records) or by athlete inquiry (questionnaires, interviews) differed in effect size.

To investigate potential publication bias, we tested whether publication status (published versus unpublished) was a significant moderator and then inspected the funnel plot and computed Egger’s regression analysis.

## Results

Across all effect sizes, the mean correlation between junior performance and senior performance was $\overline{{r} }$ = 0.148 ($\overline{{r} } ^ 2$ = 0.022, Table 2).

**Table 2:** Effects of junior performance on senior performance

	$\overline{r }$	95% CI	*p*	*k*	Comparison	*F*	*p*
Overall	0.148	0.114, 0.182	< 0.001	129			
Junior age category							
Junior A	0.215	0.174, 0.257	< 0.001	62			
Junior B	0.128	0.100, 0.156	< 0.001	45	Versus Junior A	12.216	< 0.001
Junior C	− 0.024	− 0.106, 0.057	0.515	13	Versus Junior A	34.156	< 0.001
					Versus Junior B	15.940	< 0.001
Junior D	− 0.052	− 0.101, − 0.002	0.045	9	Versus Junior A	62.586	< 0.001
					Versus Junior B	34.895	< 0.001
					Versus Junior C	0.042	0.839
Performance measure							
Dichotomous	0.140	0.078, 0.201	< 0.001	39			
Continuous	0.151	0.110, 0.192	< 0.001	90	Versus dichotomous	0.026	0.873
Performance bandwidth							
Narrow	0.178	0.146, 0.211	< 0.001	97			
Wide	0.081	0.014, 0.148	0.022	32	Versus narrow	1.119	0.292
Region							
International samples	0.183	0.150, 0.214	< 0.001	74			
National samples	0.103	0.037, 0.167	0.004	55	Versus international	0.844	0.360
Sex							
Female	0.167	0.108, 0.225	< 0.001	54			
Male	0.150	0.105, 0.194	< 0.001	67	Versus female	0.432	0.512
Types of sports							
Cgs sports	0.172	0.134, 0.210	< 0.001	88			
Game sports	0.110	0.035, 0.185	0.007	35	Versus cgs	0.175	0.676
Combat sports	0.176	− 0.365, 0.629	0.298	7	Versus cgs	0.047	0.828
					Versus game	0.125	0.724
Individual sports	0.166	0.129, 0.202	< 0.001	101			
Team sports	0.108	0.008, 0.206	0.036	29	Versus individual	0.661	0.418

$\overline{{r} }$ pooled correlation coefficient, *95% CI* confidence interval lower and upper limit, *k* number of effect sizes; *F* and *p* for moderator analyses controlling for junior age category (except among junior age categories themselves); *Junior A, Junior B, Junior C, Junior D* junior age categories, with Junior A being the oldest junior age category within each sport and Junior D the youngest age category; international samples: athletes from multiple countries, national samples: athletes from just one country, respectively; *cgs* performance is measured in centimeters, grams, or seconds (e.g., athletics, rowing, swimming, ski racing), *game* e.g., basketball, soccer, tennis, *combat* e.g., fencing, judo; *narrow* national to international competition level, *wide* regional to international competition level. The forest plots and *I*^*2*^ statistics are reported in the ESM 2, Figs. S2.1–S2.4

The effects did not significantly differ between (1) dichotomous and continuous measures of performance; (2) narrower and wider performance ranges; (3) sexes (male, female); (4) types of sports (cgs, game, and combat sports; individual and team sports); or (5) national and international samples (Table 2). The effects did differ across junior age categories: the younger the junior age category, the lower the association between junior performance and senior performance. This pattern held except for the youngest categories, Junior C and Junior D, which did not significantly differ from one another. Although effects varied across junior age categories, they were all small: junior performance explained 0–4.6% of the variance in senior performance (Table 2).

All primary studies had a high methodological quality, and risk of bias was generally low. See Table S2.1 in the ESM 2. Effects significantly differed between studies collecting performance data from public records and studies using athlete questionnaires/interviews for data collection ($\overline{{r} }$ = 0.177, 95% CI 0.145 to 0.209, versus $\overline{{r} }$ = 0.020, 95% CI − 0.037 to 0.077, *F* = 5.236, *p* = 0.024).

Of the 21 study reports, 14 were published (*k* = 106) and 7 unpublished (*k* = 23). Effect sizes did not significantly differ between published and unpublished studies (*F* = 2.367, *p* = 0.126). The funnel plot (see ESM 2, Fig. S2.5) was nearly symmetrical and Egger’s regression was nonsignificant (*b* = 9.408, 95% CI − 1.336 to 20.152, *p* = 0.086).

## Discussion

The question of whether junior performance is a valid predictor of senior performance is the subject of ongoing debate in the sports science and medicine literature. To resolve this controversy, we quantified the extent to which junior performance predicts senior performance. Specifically, we investigated the overall magnitude of the correlation between junior performance and senior performance, while testing for effects of potential moderators.

Across all effect sizes, the pooled correlation between junior and senior performance was $\overline{{r} }$ = 0.148. That is, overall, junior performance explained only 2.2% of the reliable variance in senior performance, whereas 97.8% of the variance of senior performance was explained by factors other than junior performance, along with random measurement error. The finding was robust across sexes, types of sports, dichotomous and continuous performance measures, narrower as well as wider performance ranges, and across national and international samples. Results varied across junior age categories: The younger the junior age category, the lower the association between junior performance and senior performance. However, effects were all weak. Across junior age categories, junior performance explained 0–4.6% of the variance of senior performance, meaning that 95.4–100% of the variance was explained by other factors and random measurement error.

Our finding that junior performance has little, if any, predictive validity regarding senior performance is in line with findings indicating that (1) predictors of early junior performance and of later senior performance differ and are partly opposite and (2) that successful juniors and successful seniors are largely two disparate populations [27–29]. This finding—similar to findings from other domains ([7–15], see Introduction)—has two critical theoretical implications. First, it runs counter to claims made by traditional theories of giftedness and expertise [4, 17, 18], which posit that a high level of junior performance is a prerequisite for a high level of senior performance. Second, given the weak correlation between junior performance and senior performance, the results indicate quite clearly that predictors of performance from studies among junior athletes cannot justifiably be extrapolated to senior performance. To put it another way, drawing conclusions about senior performance from research on junior performance is empirically unfounded; it may also be misleading.

From an applied perspective, talent identification and selection typically begin around puberty or younger [1, 3]—age ranges where youth performance is uncorrelated or even negatively correlated with later senior performance. Our results have major implications for policies and practices of youth sports in general and talent promotion programs in particular, in three regards. First, enhancing a youth athlete’s junior performance does not reliably lead to increased senior performance. Second, talent identification and talent promotion programs that preferentially select the highest-performing youth athletes cannot be confident they are selecting the highest future senior performers. Rather, they are selecting many individuals who will not turn out to be high performers at the senior level, while rejecting many individuals who *would* have become high performers at the senior level. Third, and relatedly, youth athletes’ current performance is not a sensitive criterion for the evaluation of talent promotion programs or of youth sport programs and youth coaches in general. The weak correlation between junior and senior performance signifies that many of the highest-performing senior athletes had low performance levels relative to many of their peers when they were juniors. That is, many of the highest-performing seniors had greater performance improvement from junior to senior age and, thus, must have developed a greater potential during junior age for long-term development through subsequent years. Therefore, a more adequate evaluation criterion of youth talent promotion is the future performance progress the youth athletes make during subsequent years into adulthood [27–29].

This study has several strengths, including a large international sample from a wide range of Olympic sports and both sexes; consideration of different junior age categories; multiple moderator analyses that showed robust findings across many different samples; and a high methodological quality of primary studies. However, several limitations should be acknowledged. First, the weak correlations found are to be interpreted as associations, not causal links. Second, male samples and national samples from Western European countries were over-represented. Third, primary studies involved athletes from regional to international competition levels, but no athletes below these levels. It may be that associations between junior and senior performance vary among lower-performing populations or populations extending over wider performance ranges. Fourth, successful juniors who withdrew from competitive sports before reaching senior age, as well as successful seniors who did not begin competing within junior age categories, may not have been considered in primary studies. Finally, although we used multiple databases, bias related to availability of study reports, location of study (i.e., country), and language of study report is possible, as in any systematic review.

Future research should seek to extend investigations to populations that have previously been under-represented, including athletes from countries other than Western Europe, Paralympic sports, and female athletes. Furthermore, future researchers should investigate whether multivariate linear and nonlinear interactions between youth performance and factors associated with athletes’ childhood/adolescent participation patterns, coaching, practice characteristics, relative age, timing of biological maturation, health, basic physical abilities and perceptual-motor skills, social support, and psychological characteristics provide better predictive value than youth performance alone. On a final note, rather than extrapolating from junior samples, theories concerning predictors of the highest senior performance levels should be based on empirical findings from the highest-performing senior athletes themselves. This research will improve the quality of the available empirical evidence, foster the advancement of even better theories of the acquisition of athletic expertise, and thereby provide the empirical substantiation needed for evidence-based practical recommendations.

### Supplementary Information

Below is the link to the electronic supplementary material.

Supplementary file1 (XLSX 103 KB)

Supplementary file2 (DOCX 1494 KB)

## Footnotes

1 A recent systematic review [27] synthesized studies that investigated at a collective level the number of athletes (i.e., proportions of samples) who did or did not achieve an equivalent competition level within both junior and senior age categories. Based on a separate data set, the present meta-analysis extends this previous investigation by investigating the correlation between junior and senior performance, i.e., quantifying at the individual athlete level the extent to which individual differences in junior performance predict individual differences in senior performance.

## Declarations

### Data availability

All original data are freely available in the electronic supplementary material 1.

### Code availability

Code for data cleaning and analysis is available from the corresponding author on reasonable request.
