# Supplementary material for: Quantifying the Extent to Which Junior Performance Predicts Senior Performance in Olympic Sports: A Systematic Review and Meta-analysis
Source: Sports Med. 2023 Sep 7;54(1):95–104. doi: 10.1007/s40279-023-01906-0 (PMC10799111; doi:10.1007/s40279-023-01906-0)
Supplement: Supplementary file 2 — Supplementary file2 (DOCX 1494 KB) [file 40279_2023_1906_MOESM2_ESM.docx]

**Quantifying the extent to which junior performance predicts senior performance in Olympic sports: A systematic review and meta-analysis**

Michael Barth, Arne Güllich, Brooke N. Macnamara, David Z. Hambrick

Electronic supplementary material (ESM) 2

Table S2.1 Quality of the primary studies using the Mixed Methods Appraisal Tool (MMAT).

| No | Authors | Year | MMAT 1 Are the participants representative of the target population? | MMAT 2 Are measurements appropriate regarding both the outcome and intervention (or exposure)? | MMAT 3 Are there complete outcome data? | MMAT 4 Are the confounders accounted for in the design and analysis? |
| --- | --- | --- | --- | --- | --- | --- |
| 1 | Barreiros & Fonseca | 2012 | yes | yes | yes | yes |
| 2 | Barth et al. | 2019 | yes | yes | yes | yes |
| 3 | Bjoerndal et al. | 2018 | yes | yes | yes | yes |
| 4 | Brouwers et al. | 2010 | yes | yes | yes | yes |
| 5 | Brustio et al. | 2021 | yes | yes | yes | yes |
| 6 | Güllich | 2018 | yes | yes | yes | yes |
| 7 | Güllich | 2019 | no | yes | yes | yes |
| 8 | Güllich | 2014a | yes | yes | yes | yes |
| 9 | Güllich | 2014b | yes | yes | yes | yes |
| 10 | Güllich & Emrich | 2013 | yes | yes | yes | no |
| 11 | Güllich & Emrich | 2014 | yes | yes | yes | yes |
| 12 | Güllich et al. | 2019 | yes | yes | yes | no |
| 13 | Güllich et al. | 2009 | yes | yes | yes | yes |
| 14 | Hardy et al. | 2013 | yes | yes | yes | yes |
| 15 | Hornig et al. | 2016 | yes | yes | yes | yes |
| 16 | Kalén | 2017 | yes | yes | yes | yes |
| 17 | Kristiansen & Stensrud | 2020 | no | yes | yes | yes |
| 18 | Reid et al. | 2007 | yes | yes | yes | yes |
| 19 | Schuhmacher et al | 2007 | yes | yes | yes | yes |
| 20 | Sigmund & Güllich | 2021 | yes | yes | yes | yes |
| 21 | Zsombor et al. | 2020 | yes | yes | yes | yes |

For further criteria explanations see Hong QN, Pluye P, Fàbregues S, Bartlett G, Boardman F, Cargo M, et al. Mixed Methods Appraisal Tool (MMAT): version 2018: user Guide; 2018. http://mixedmethodsappraisaltoolpublic.pbworks.com/w/file/fetch/127916259/MMAT_2018_criteria-manual_2018-08-01_ENG.pdf. Accessed 23 Jul 2022.


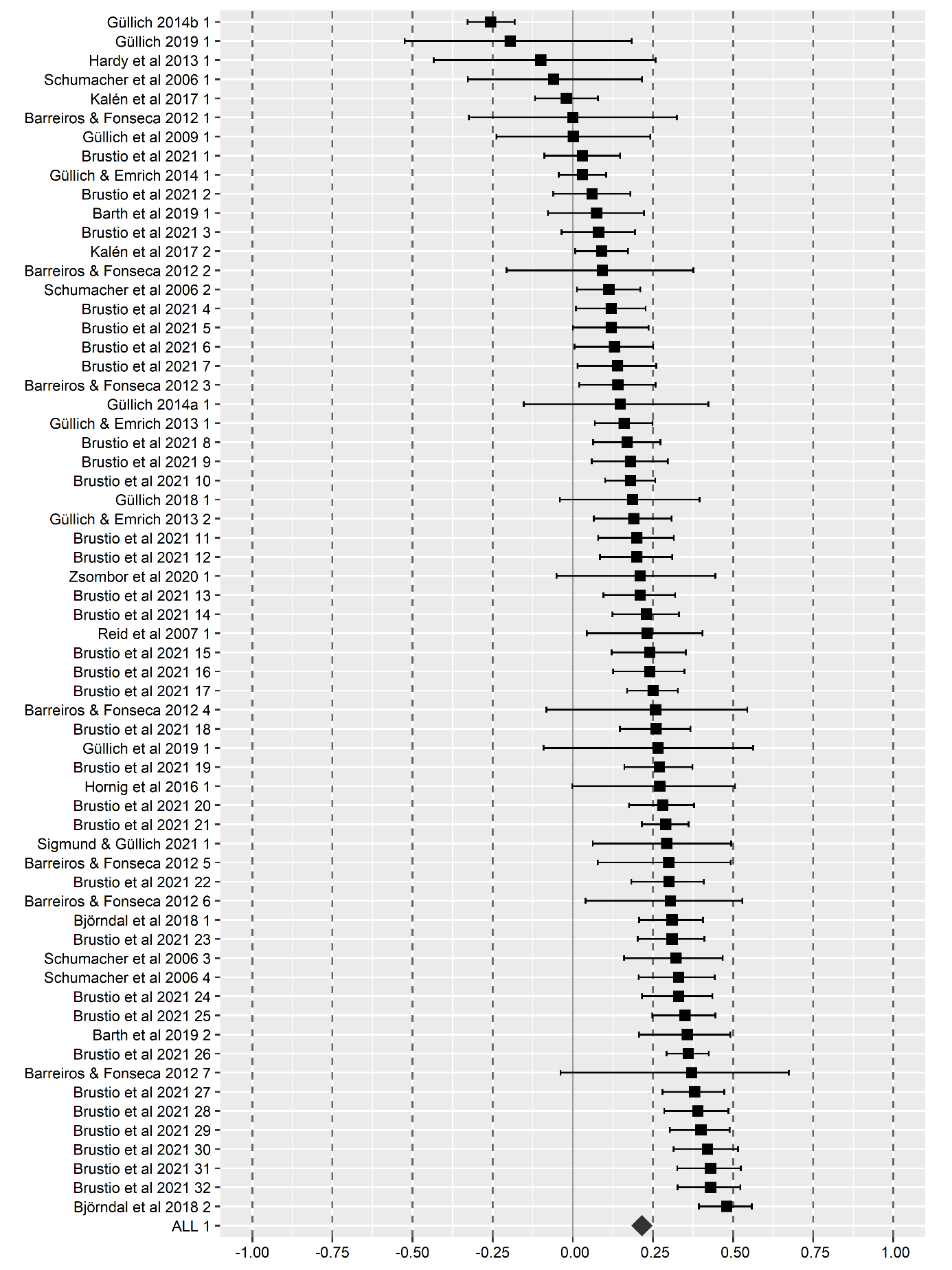


Figure S2.1 Correlation coefficients between the highest junior age category (Junior A) and senior success. Forest plot depicting each effect size (squares) and its 95% confidence interval (lines) entered into the meta-analytic model for correlations between success in Junior A and senior success, along with the meta-analytic mean effect size (diamond). The dataset is the dataset for the estimation of the overall pooled correlation coefficient, but now also including outliers. The numbers are incidences of subsamples. *I^2^*=82.38.


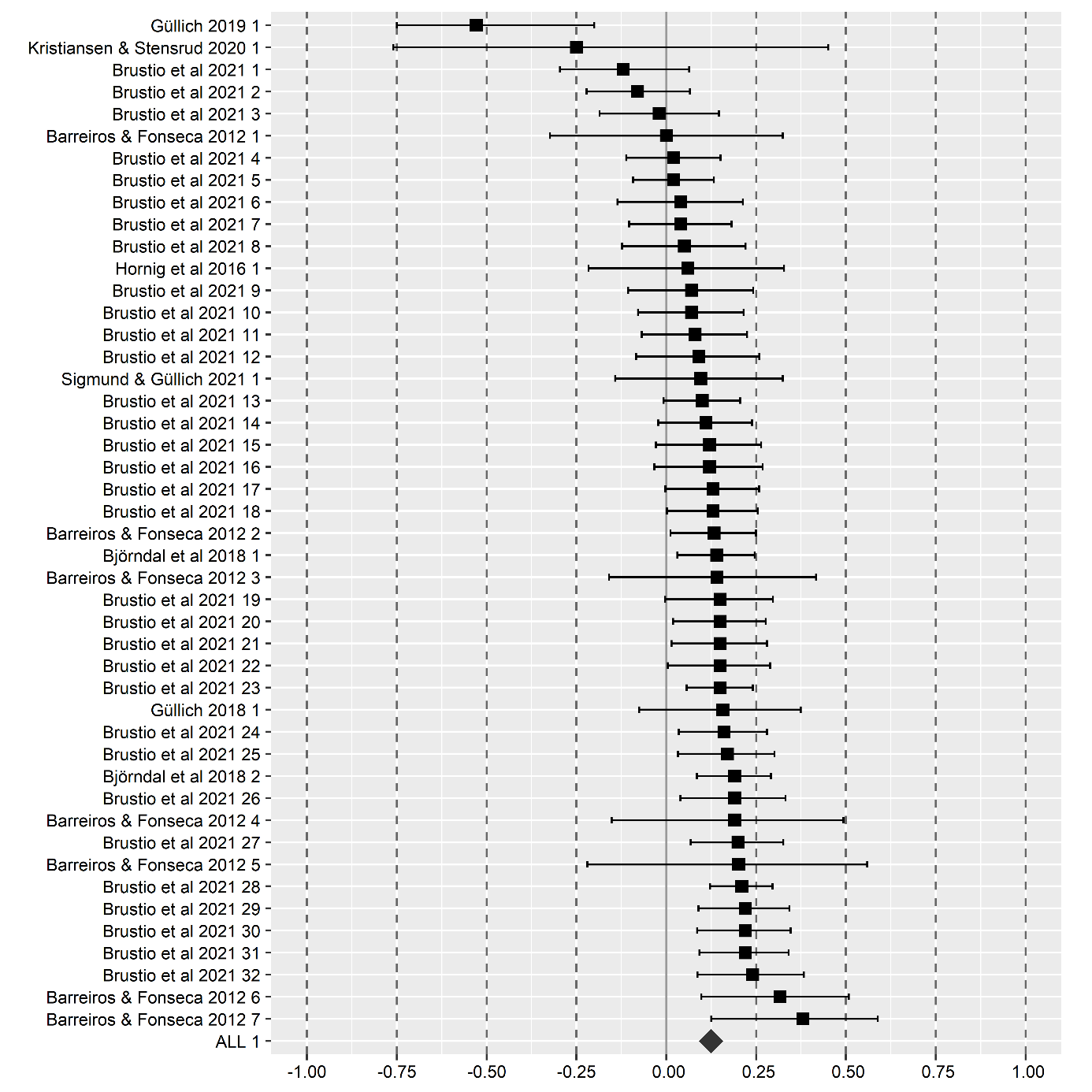


Figure S2.2 Correlation coefficients between the second highest junior age category (Junior B) and senior success. Forest plot depicting each effect size (squares) and its 95% confidence interval (lines) entered into the meta-analytic model for correlations between success in Junior B and senior success, along with the meta-analytic mean effect size (diamond). The dataset is the dataset for the estimation of the overall pooled correlation coefficient, but now also including outliers. The numbers are incidences of subsamples. *I^2^*=21.45.


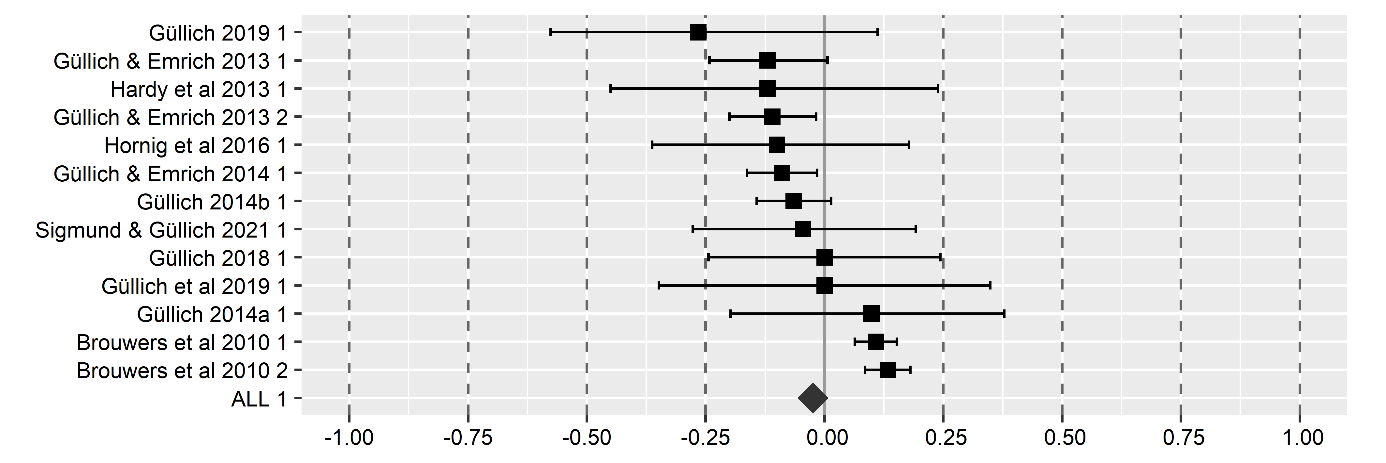


Figure S2.3 Correlation coefficients between the second lowest junior age category (Junior C) and senior success. Forest plot depicting each effect size (squares) and its 95% confidence interval (lines) entered into the meta-analytic model for correlations between success in Junior C and senior success, along with the meta-analytic mean effect size (diamond). The dataset is the dataset for the estimation of the overall pooled correlation coefficient, but now also including outliers. The numbers are incidences of subsamples. *I^2^*=77.08.


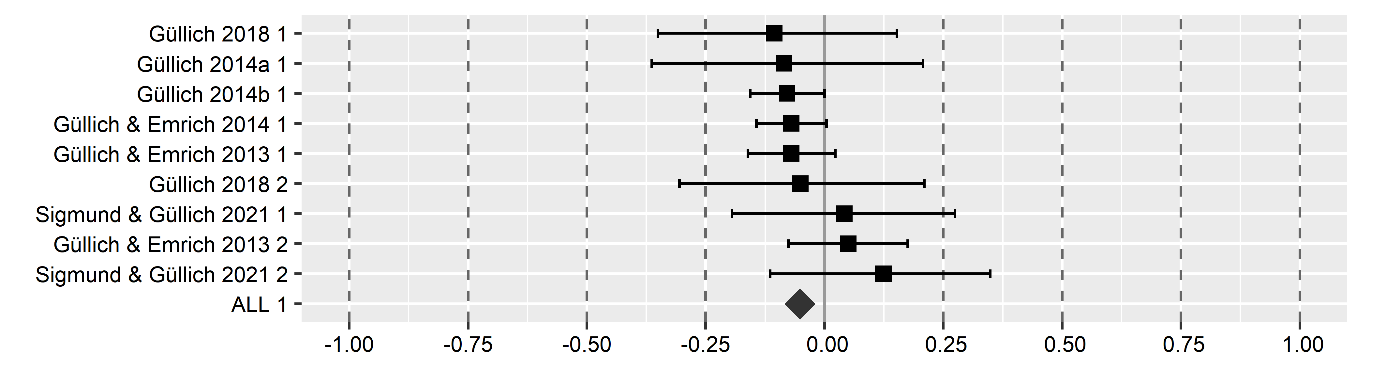


Figure S2.4 Correlation coefficients between the lowest junior age category (Junior D) and senior success. Forest plot depicting each effect size (squares) and its 95% confidence interval (lines) entered into the meta-analytic model for correlations between success in Junior D and senior success, along with the meta-analytic mean effect size (diamond). The dataset is the dataset for the estimation of the overall pooled correlation coefficient, but now also including outliers. The numbers are incidences of subsamples. *I^2^*=0.00.


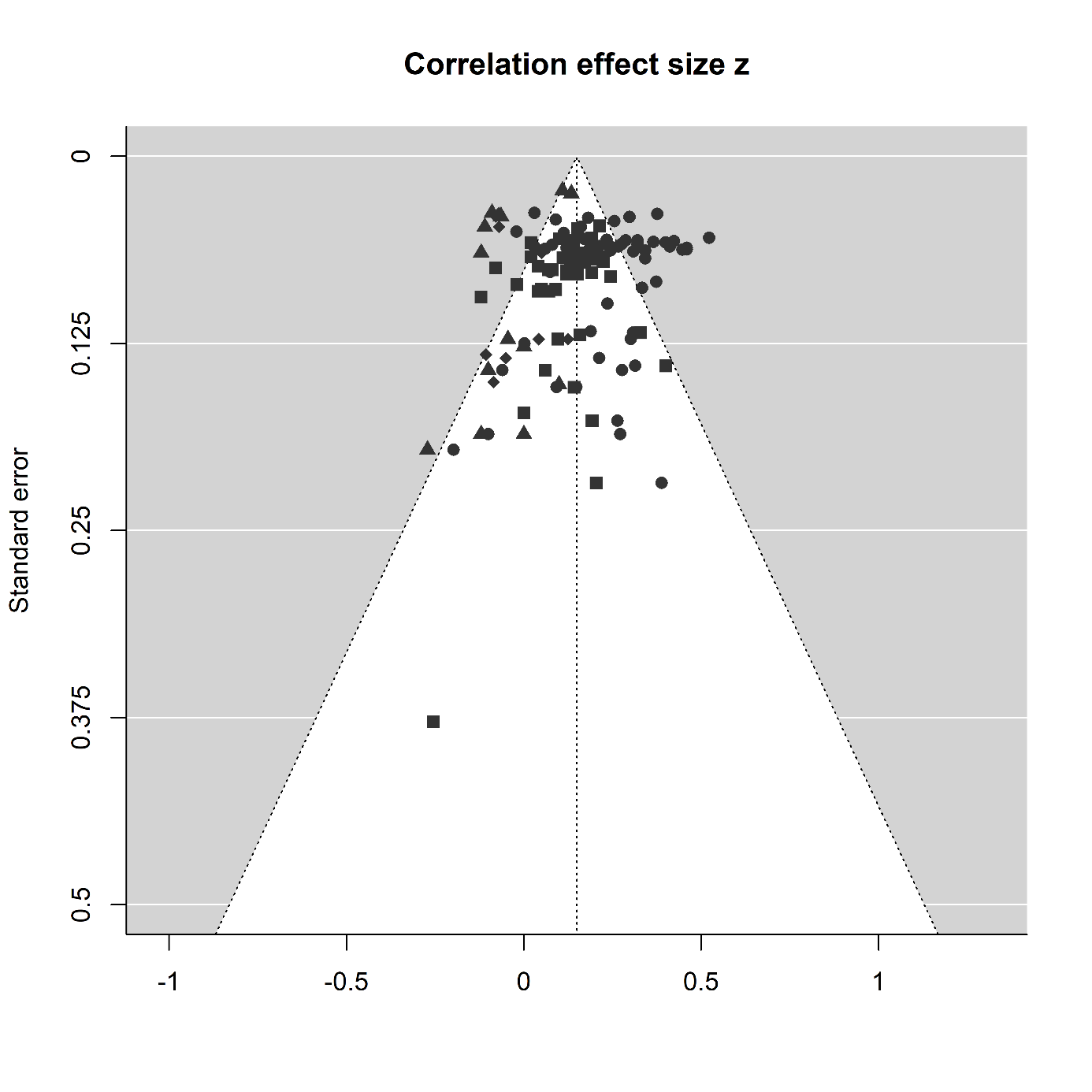


Figure S2.5 Funnel plot (with Junior A ●, Junior B ■, Junior C ▲, Junior D ♦)

# References

1. Barreiros AN, Fonseca AM. A retrospective analysis of Portuguese elite athletes' involvement in international competitions. Int J Sports Sci Coach. 2012;7:593–600. https://10.1260/1747-9541.7.3.593

2. Barth M. Analysing international senior success in alpine ski racing: success concentration and predictability: Unpublished manuscript, University of Innsbruck.; 2019.

3. Bjørndal CT, Luteberget LS, Holm S. The relationship between early and senior level participation in international women's and men's handball. J Hum Kinet. 2018;63:73–84. https://10.2478/hukin-2018-0008

4. Brouwers J, De Bosscher V, Schaillee H, Truyens J, Sotiriadou P. The relationship between performances at U-14 international youth tournaments and later success in Tennis. Medicine and Science in Tennis. 2010;15:21–5.

5. Brustio PR, Cardinale M, Lupo C, Varalda M, Pasquale P de, Boccia G. Being a top swimmer during the early career is not a prerequisite for success: a study on sprinter strokes. J Sci Med Sport. 2021;24:1272–7. https://10.1016/j.jsams.2021.05.015

6. Guellich A, Seiler S, Emrich E. Training methods and intensity distribution of young world-class rowers. Int J Sports Physiol Perform. 2009;4:448–460 (unpublished data from the study). https://10.1123/ijspp.4.4.448

7. Güllich A. Many roads lead to Rome—developmental paths to Olympic gold in men's field hockey. Eur J Sport Sci. 2014a;14:763-71 (unpublished data from the study). https://10.1080/17461391.2014.905983

8. Güllich A. Selection, de-selection and progression in German football talent promotion. Eur J Sport Sci. 2014b;14:530–7. https://10.1080/17461391.2013.858371

9. Güllich A. Sport-specific and non-specific practice of strong and weak responders in junior and senior elite athletics - a matched-pairs analysis. J Sports Sci. 2018;36:2256–64 (unpublished data from the study). https://10.1080/02640414.2018.1449089

10. Güllich A. "Macro-structure" of developmental participation histories and "micro-structure" of practice of German female world-class and national-class football players. J Sports Sci. 2019;37:1347–1355 (unpublished data from the study). https://10.1080/02640414.2018.1558744

11. Güllich A, Emrich E. Investment patterns in the careers of elite athletes in East and West Germany. European Journal for Sport and Society. 2013;10:191–214. https://10.1080/16138171.2013.11687919

12. Güllich A, Emrich E. Considering long-term sustainability in the development of world class success. Eur J Sport Sci. 2014;14 Suppl 1:S383-97 (unpublished data from the study). https://10.1080/17461391.2012.706320

13. Güllich A, Hardy L, Kuncheva L, Woodman T, Laing S, Barlow M, et al. Developmental biographies of Olympic super-elite and elite athletes: a multidisciplinary pattern recognition analysis. Journal of Expertise. 2019;2.

14. Hardy L, Laing S, Barlow M, Kincheva L, Evans L, Rees T, et al. A comparison of the biographies of GB serial medal and non-medalling Olympic athletes. London; 2013.

15. Hornig M, Aust F, Güllich A. Practice and play in the development of German top-level professional football players. Eur J Sport Sci. 2016;16:96–105 (unpublished data from the study). https://10.1080/17461391.2014.982204

16. Kalén A, Pérez-Ferreirós A, Rey E, Padrón-Cabo A. Senior and youth national team competitive experience: influence on player and team performance in European basketball championships. Int J Perform Anal Sport. 2017;17:832–47. https://10.1080/24748668.2017.1405610

17. Kristiansen E, Stensrud T. Talent development in a longitudinal perspective: Elite female handball players within a sport school system. Transl Sports Med. 2020;3:364–73. https://10.1002/tsm2.143

18. Reid M, Crespo M, Santilli L, Miley D, Dimmock J. The importance of the International Tennis Federation's junior boys' circuit in the development of professional tennis players. J Sports Sci. 2007;25:667–72. https://10.1080/02640410600811932

19. Schumacher YO, Mroz R, Mueller P, Schmid A, Ruecker G. Success in elite cycling: a prospective and retrospective analysis of race results. J Sports Sci. 2006;24:1149–56. https://10.1080/02640410500457299

20. Sigmund P, Güllich A. Individualisation, readjustment and athlete codetermination of high-performance training in athletics and volleyball. Int J Sports Sci Coach. 2021:1–10 (unpublished data from the study). https://10.1177/17479541211043183

21. Zsombor Z, Ágoston N, Tamás S. Competition experience, relative age effect and average age of the senior world events’ medal-winning basketball players. Studia Educatio Artis Gymnasticae. 2020;65:5–18. https://10.24193/subbeag.65(3)
